# Community, system and policy level drivers of bovine tuberculosis in smallholder periurban dairy farms in India: a qualitative enquiry

**DOI:** 10.1186/s12889-019-6634-3

**Published:** 2019-03-13

**Authors:** Abhimanyu Singh Chauhan, Mathew Sunil George, Johanna Lindahl, Delia Grace, Manish Kakkar

**Affiliations:** 1 Public Health Foundation of India, Plot 47, Sector 44, Gurgaon, Haryana 122002 India; 2 Department of Public Health Sciences, Faculty of Medicine, University of Liège - Hospital District, Hippocrates Avenue 13 - Building 23, 4000 Liège, Belgium; 3 Indian Institute of Public Health, Gurgaon, Haryana 122002 India; 4 Centre for Research and Action in Public Health (CeRAPH), University of Canberra, Building 22, Floor B, University Drive, Bruce, ACT 261 Australia; 5 International Livestock Research Institute, Nairobi, 30709-00100 Kenya; 6 Zoonosis Science Laboratory, Uppsala University, Po Box 582, SE-751 23 Uppsala, Sweden; 7 Department of Clinical Sciences, Swedish University of Agricultural Sciences, PO Box 7054, SE-750 07 Uppsala, Sweden

**Keywords:** Bovine tuberculosis, Dairy farm, Dairy farmers, Qualitative, Zoonoses, India

## Abstract

**Background:**

Rapid urbanization has led to expansion of peri-urban fringes, where intensive, industry-style livestock rearing has led to emerging vulnerabilities at the human-animal-environment interface. This study was undertaken to understand the health system and farm-level factors that influenced the risk of transmission of bovine Tuberculosis (bTB) in animals and humans in peri-urban smallholder dairy farms of India.

**Methods:**

Thematic guides were developing through literature review and expert consultation. In-depth interviews were conducted till attainment of saturation. Identification of core themes was followed by etiological enquiry and generation of a conceptual model.

**Results:**

Veterinarians were consulted as a last resort after home-remedies and quacks had failed. Damage control measures, especially with respect to- selling or abandoning sick animals, added to the risk of disease transmission. Although civic authorities believed in the adequacy of a functioning laboratory network, end users were aggrieved at the lack of services. Despite the presence of extension services, knowledge and awareness was limited, promoting risky behaviour. The absence of cogent policies in dealing with bTB was a significant barrier. Stakeholders did not consider bTB to be a major concern. It is possible that they underestimate the problem.

**Conclusion:**

The current study helps to identify gaps which need to be addressed through collaborative research, and OneHealth interventions to build community awareness.

**Electronic supplementary material:**

The online version of this article (10.1186/s12889-019-6634-3) contains supplementary material, which is available to authorized users.

## Background

Bovine tuberculosis (Btb) is a disease of infectious nature that occurs in cattle and can spread to humans by aerosol inhalation or ingestion of unpasteurized milk [1, 2]. *Mycobacterium bovis*, the cause of bovine tuberculosis, has been identified in humans in most countries where isolates of mycobacteria from human patients have been fully characterized. The incidence of pulmonary tuberculosis caused by *M. bovis* is higher in occupationally exposed individuals such as farm and slaughterhouse workers than in urban inhabitants [1]. The transmission of *M. bovis* to humans via milk and its products is eliminated by the pasteurization of milk. Btb has been included in the list of notifiable terrestrial and aquatic animal diseases as given by OIE [3]. In developed nations, the disease has ceased to be a public health problem owing to effective implementation of robust prevention and control measures. However, in India the epidemiology of Btb still remains poorly characterized. Although bTB has been identified as a priority zoonosis in India [4], little is known about disease transmission from human to cattle and vice-versa, risk factors and mechanisms to prevent the same. Even the Revised National Tuberculosis Control Program (RNTCP), fails to distinguish between TB of human (*M. tuberculosis*) and animal (*M. bovis*) origin [5]. Both mycobacteria can infect both humans and cattle, but we reserve the term bTB for the disease caused by *M. bovis*, in difference to meaning any tuberculosis occurring in bovines. A recent systematic review by Muller and colleagues has highlighted the existing gaps in basic epidemiological data relating to Btb globally with special emphasis to South East Asian region [6]. Reported prevalence of Btb in India varies from 1.6 to 51.2% in cattle [7–9]. With agricultural intensification and practice of herding together of (highly susceptible) exotic, crossbred animals, the risk of emergence and spread of Btb is bound to escalate [10]. Considering the projections related to increased intensification of dairy farming and the resultant increase in the probability of zoonotic transmission, recent meta-analysis by S Srinivasan et at suggested that to eradicate human tuberculosis, a parallel effort is required in controlling bTB in cattle population in India [9].

The current study is part of larger initiative with a focus on periurban smallholder dairy farms. Smallholder dairy farming, typical of periurban ecosystems, suffers from lack of support and quality control of dairy farming as well as the absence of an organized system of farm inspection or screening of animals for disease [11]. Agricultural intensification in these settings thus poses significant public health risks, including the potential for zoonotic disease transmission and emergence of new diseases [12, 13]. This qualitative enquiry was conducted to gain understanding into dairy farmers’ community and health system and policy level factors that could influence Btb transmission in these dairies.

## Methodology

### Study setting

This qualitative inquiry was undertaken among smallholding dairy farmers in peri-urban areas of three cities of India i.e. Guwahati, Ludhiana and Bangalore. In general, periurban zones of a typical city comprise a wide range of activities, including harvesting, animal husbandry, real estate investments, housing development and waste dumping etc. [11]. Description of a periurban area is not well documented and varies between region to region and country to country, making it difficult to assess the precise number of inhabitants in the fringe. A large section of population from rural to urban movement settle down in periurban bounds of cities [11]. Thematic guides were prepared through literature review and expert consultation (Additional file 1). Data collection was done in the months of February 2015 to January, 2016.

### Sampling and data collection

Respondents were identified through purposive sampling with the help of officials at local veterinary colleges and Non-Government Organization (NGO) partners. Snowballing technique was used to identify and ascertain stakeholder relevant for the study [14]. No new enrolments or interviews were conducted once the data collected reached saturation in terms of new emerging information across themes.

The interviews were conducted by MSG (male) and ASC (male). Both interviewers were practicing public health investigators with significant experience in community processes, participatory methods and field level qualitative data collection methods. Interviewers held postgraduate degrees in public health with specialisation in qualitative field data collection methods. Face-to-face interviews with identified stakeholders were conducted at a place convenient to them. Partnering NGOs assisted setting up of schedules for interaction at a time convenient to the respective stakeholder. Most interviews with community level stakeholders were done in their respective local languages and most health systems and policy level officials were interviewed in English. On average, an IDI lasted nearly for 1 h. All interviews were tape-recorded, transcribed and translated from local language into English by professional agency, and double-checked against original tape recordings.

### Data management and analysis

Content analyses was used to undertake the data analysis. Software package AtlasTi 7.2® was used to code the transcripts, utilizing a reflexive and inductive approach to allow codes and categories to emerge from within the data. Coding was done by two investigators (MSG and ASC) and disagreements were addressed in discussion with senior investigators (MK and DG).

An iterative process ensured that the data collected was grounded and have rich details related to the topic of inquiry [14].

### Quality assurance

Interviews were conducted by qualified researchers. Interviews were scrutinised for completeness, correctness, and transcription and translation of responses with proper tagging of recordings. About 30% of the interviews from every study site were randomly verified for their correct transcription and translation. Due to inherent limitations of interpretation of qualitative data from culturally diverse as well from different farming systems, the data collection team undertook regular meetings with the steering group [14]. The study is compliant with the consolidated criteria for reporting qualitative research (COREQ) for reporting findings of the qualitative research study [15].

## Results

Details of the stakeholders interviewed under this study is provided under Table 1. As the study included three study sites, region specific participants were identified through a snowballing process.

**Table 1:** Details of the stakeholders interviewed

Study site/Stakeholders	Dairy farmer	Veterinary/Extension officer	Veterinary field assistant	Trader	Pharmacist/Drug distributor	Civic or union official
Guwahati	7	5	3	3	3	3
Bangalore	4	6	2	N/A	2	3
Ludhiana	4	2	2	N/A	2	3

The results are categorised as three core themes:

1. Knowledge and practices related to Btb
2. Limited system support for prevention and control of Btb
3. Lack of effective policies and programmatic direction in the context of Btb

Core theme are further divided into themes and sub-themes. These are listed in Table 2.

**Table 2:** Theme emerged in context to Btb in periurban areas in India

Sl. No.	Domain	Core themes	Sub-themes
1	Community and Individual	Inadequate knowledge and practices related to Btb	Lack of knowledge of Btb in cattle and its zoonotic potential
			Limited evidence on Btb
			Absence of screening of cattle for Btb during purchase
2	Veterinary health system support	Limited system support for prevention and control of Btb	Absence of laboratory support to diagnose Btb and make informed decision for prevention and control
			Inadequate support from extension services
			Lack of technical and operational guidance on disposal of infected and dead animals
3	Policy and market scenario	Lack of effective policies and programmatic direction in the context of Btb	Limited focus on Btb under current livestock health programs in India
			Lack of Btb specific surveillance and response system
			Limited guidance on treatment protocol/procedure to deal with Btb

Systems concept allowed to study the associations and interplay between the sub-themes and core themes that functions at different levels. Levels are explained in detail in a previous publication associated with the larger initiative [14].

### Core theme I: Knowledge and practices related to BTB

Limited knowledge and practices related to Btb, especially among farmers, emerged as an important core theme. This was further determined by lack of understanding of disease causation, transmission, prevention and control, animal and public health impacts; availability of limited evidence to support attention by local stakeholders which reflected in absence of screening of cattle for Btb.

### Sub-theme one: Lack of knowledge of Btb in cattle and its zoonotic potential

#### Dairy farmers’ knowledge

Farmers could only relate the word ‘tuberculosis’ to human. A strong construct of ‘Tb only in human’ was reflected from the interviews. Farmers were mainly aware about the clean milk production and prevention & control of mastitis, brucellosis and foot & mouth disease. Due to lack of awareness about the possibility of cattle suffering from Btb, knowledge related to prevention and control was universally absent. Interestingly, few farmers could also tell about treatment of human tuberculosis. Nearly half the farmers were aware about brucellosis and its zoonotic potential. Among those who were aware, they knew that brucellosis can transmit between animal-animal and human-animal.

Veterinarians demonstrated good understanding of Btb, its zoonotic potential, symptoms and, prevention and control measures. However, veterinary field assistants at all three sites had limited understanding of Btb. Most were aware about tuberculosis in cattle but not sure about zoonotic potential.

### Sub-theme two: Limited evidence with veterinary health system on Btb

Veterinary official’s perspective: According to state officials and veterinarians there is absence of estimates on burden of Btb in cattle population. As a result, the disease holds less importance for policy makers due to absence of evidence. Many veterinarians reported having come across clinical symptoms of Btb in cattle during their field visits. Few veterinarians also reported signs of tuberculosis infection during post mortem examination of cattle. One pathologist reported that he had recently come across cases of tuberculosis in cattle while conducting autopsies in one of the sites. He felt that this could potentially be an important area to study as nothing is known about the extent of the problem.

“I think bTB is of great importance now but we don’t have much data on it. Maybe you can study this especially in *goshalas*, where older cattle are kept. All the six cases I came across last year were from a *goshala* and they did not know it was tuberculosis. It was only when post mortem was done did we come across nodules suggestive of *M. bovis* infection.” Pathologist - Government

### Sub-theme three: Absence of screening of cattle for Btb during purchase

#### Dairy farmers’ opinion

Screening of animals for diseases is almost universally done only by visual inspection. Farmers have been taught traditionally that the gait of the animal and its posture helps identify which animal is healthy and which is diseased. However, farmers also admitted pitfalls of this approach and the fact that there were occasions when they had managed to sell their animals that were sick at fairs.

“We generally go to the fair and observe the physical characteristics of the animals on sale and how it walks how it stands etc. Based on that we decide whether to buy the animal or not.” Dairy farmer

Veterinarians also admitted that in general, farmers did not screen animals before buying them and that the practice of routine screening of animals to detect any infections was practically non-existent. Similarly, sick cattle are often sold in cattle fairs.

“Actually, we don’t have any policy to screen for TB in animals. Nothing has been done in this regard. Sometimes when there are studies carried out we have found cases, but there was no policy on what to do so it was left at that.” Veterinarian

### Core theme two: System level support for prevention and control of Btb

Limited systems support, outreach and oversight for prevention and control of Btb emerged as a core theme at the veterinary health systems level. This core theme could be further explained based on the following factors: inadequate support of extension services, lack of laboratory support to diagnose Btb and make informed decision for prevention and control and lack of technical and operational guidance on disposal of infected and dead animals.

### Sub-theme one: Laboratory support to diagnose Btb

#### Government officials’ perspective

Officials from the animal husbandry department reported operationally functional labs and diagnostic support services to the field veterinarians. However, when asked specifically about Btb, very few veterinarians mentioned about the skin testing facility being available at the army farms or progressive dairy farms having large holdings. Routine screening as well as diagnostic facility for bovine tuberculosis in cattle for smallholder dairy farms was reported to be universally absent.

#### Field veterinarians’ perspective

Veterinarians reported that they are not dependant on laboratory results to treat cattle in the field. Treatment is mostly based on case history and symptomatic assessments. Veterinarians mentioned that in most of the cases the farmers seeks veterinarian’s consultation when cattle is critically ill after being all self-treatment and drug administration is tried, and resulted in no improvement.. Veterinarian immediately has to attend the cattle to prevent the loss of life. Secondly, according to the field veterinarians, the laboratories are not fully equipped nor completely functional. Therefore, even if they want to access the services, they do not receive the desired response both in terms of timeliness and quality. No veterinarian reported to have ever accessed laboratory facilities for testing Btb.

“If the lab is in working condition we don’t have a microbiologist, if the microbiologist is there then there is no proper equipment. So how do I make use of it? On paper it is all there but practically it is not possible. If I need a lab report, then I ask them to go to the university or to some private labs to get a report.” Veterinarian

“Look we treat primarily from the case history of the sick animal and after some years of experience you know that this animal is in this condition means it is suffering from this problem and this is the treatment. Other than that not much.” Veterinarian

### Sub-theme two: Inadequate support from extension services

#### Dairy farmers’ perspective

It’s noteworthy that none of the dairy farmers in the study locations mentioned being benefitting from the extension services and all perceived them as substandard quality both in terms of content and delivery. They also reported that the extension services were undertaken as field level training to the university students. None of the farmers mentioned about any information being given specifically on Btb.

“What services are you talking about? There is such a big college here and they can’t even provide us with proper semen.” Dairy farmer union official

“No we do not get anything from the department or college.” Dairy farmer

“The department does organise activities from time to time when they want to train their students. Other than that, such activities are not focused on small farmers and their farms.” Dairy farmer

#### Extension department officials’ opinion

Extension units are working efficiently and offers services to local dairy farmers on a consistent basis. Most of these extension services were presented free of charge so that all dairy farmers in these areas could avail them. Across the study locations, information being given to dairy farmers on farm hygiene and clean milk production was reported. None of the officials mentioned about any information session mentioning Btb.

“Regular meetings are organized by the department and we have sessions taken by experts to give them the latest know-how on various issues related to management of a dairy farm.” Senior Extension department official

Many veterinarians reported that majority of the small holding dairy farmers do not to join the workshops organised by extension department. According to them, this is due to the farmers’ confidence on traditional knowledge which they receive via Intergenerational transfer of knowledge and practices in context to animal husbandry. On the other hand, new progressive farmers are relatively keen for learning the latest methods and developments in the field of dairy farming, and also receptive to the behavioural modifications.

### Sub-theme three: Lack of technical and operational guidance on disposal of dead cattle

#### Dairy farmers’ perspective

The farmers reported not receiving any regular formal support for disposal of dead animals. Disposal was organized by the farmers themselves which came at a cost and added to losses incurred on account of treatment as well as loss of productivity. As a result, farmers preferred selling animals when sick and not responsive to treatment.

“If any of our animals die then we have to pay to get someone to come and remove the body (carcass) and take it away. We don’t get any support for this from anyone. So we don’t keep very sick animals here.” Dairy farmer

Dairy farmers referred to a specific community of people who visited farms and took sick animals away. In some sites they were referred to as ‘Mohammadeans’. Members of this community visit dairy farms on a regular basis, eyeing cattle, which showed signs and symptoms of any illness and offering prices based on the condition and age of the cows or buffaloes. While we did not get the opportunity to meet with any member of this community, dairy farmers across the three sites considered them as their allies (different local names) as they helped in easy disposal of sick animals. However, farmers remained unaware about the fate of this cattle.

### Core theme three: Programme and policy context of Btb

Lack of effective policies and programmatic direction in the context of Btb emerged as the core theme at policy level. The policy context of Btb could be further explained based on three sub themes that emerged inductively, namely limited focus on Btb under current livestock health programs, lack of Btb specific surveillance and response system and limited guidance on treatment protocol/procedure to deal with Btb.

### Sub-theme one: Limited focus on Btb under current livestock health programs in India

Assistance to States for Control of Animal Diseases (ASCAD) as a federally funded program provides provision for inclusion of diseases deemed important by the state. However, senior officials reported that there is no separate initiative to address Btb including, under ASCAD. It was also reported that limited screening of animals, as a diagnostic procedure, is done in veterinary colleges or on request by farmers in a more organized dairy farming set up.

### Sub-theme two: Lack of Btb specific surveillance and response system

Routine surveillance was reported to be universally absent and no systems were in place to obtain any data in this regard. It was only during outbreaks when animal husbandry department and other officials got involved in collecting information about various cattle in the area and if any of them were infected with the particular disease of interest that had caused the outbreak (e.g. Foot and Mouth disease, etc.). Efforts are primarily centred on outbreak and once the issue had settled down surveillance and screening of animals also tended to stop. However, it has never been done for Btb.

“When we had an outbreak of FMD then they came to screen all the animals. Otherwise nothing” Veterinarian

“We do not have any data and that is our biggest problem. First of all, we need to collect proper data on the animals that we have without that we can’t progress.” Senior government official

### Sub-theme three: Limited guidance treatment protocol/procedure to deal with Btb

#### Veterinarians’ opinion

Extension department officials in one of the sites pointed out that some years ago cases were found in animals held at the local veterinary university but there was no policy regarding what was to be done after that. A senior official pointed out that there was neither a policy about what to do with Btb nor adequate facilities to test samples.

“Actually we don’t have any policy to screen for TB in animals. Nothing has been done in this regard. Sometimes when there are studies carried out we have found cases, but there was no policy on what to do, so it was left at that.” Veterinarian

“See I am able to detect and find brucellosis why because there is support under a national programme. It is only after that I am able to test many samples and find out otherwise it is very difficult to do this work. So for TB so far we have nothing.” Senior official, Government Lab

#### Farmers’ perspective

Farmers were unaware of Btb as a disease that affected cattle and had marked impact on health and productivity of animals. However, as elicited in disease management practices, once some unsuccessful attempts have been made to treat the animals, a sick cow is often sold to minimize losses. This should include cows suffering from Btb which typically results in disease signs/symptoms such as cough, wasting and loss of productivity in cows.

There is a significant variation between the three selected study sites in context to dairy farming systems. Guwahati dairy farming system is unregulated and mainly dominated by traders. In contrast, Bangalore and Ludhiana are fairly under influence of cooperative unions which are relatively active in providing information services etc. related to disease transmission and animal health. Dairy system could potentially be a driving force affecting farm level disease dynamics and non-prescribed usage of veterinary antimicrobials.

### Conceptual model

**Fig. 1:**
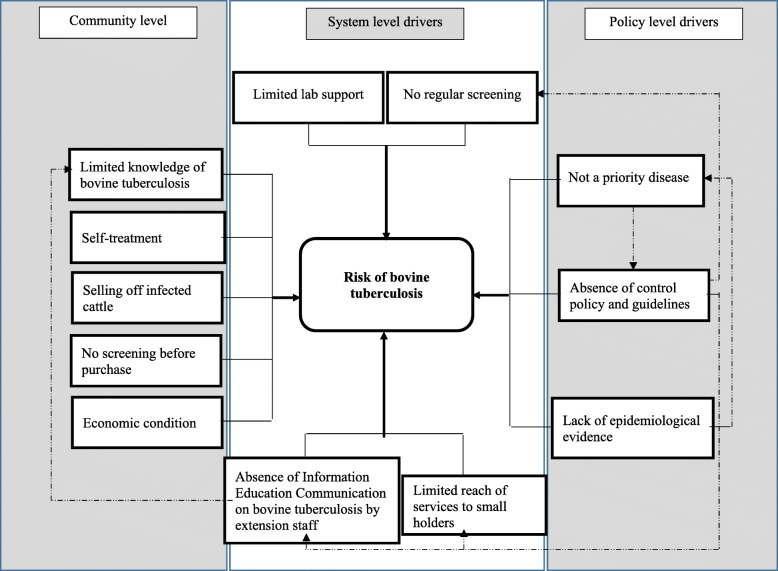
Conceptual model showing the interplay of potential drivers contributing to transmission of bovine tuberculosis in peri-urban smallholding dairy farms in India

A conceptual model was developed to present the findings of the study. The potential drivers contributing to the transmission of Btb in periurban small holding dairy farms are categorized into community, health system and policy level. This was done considering the level there are operating in. Additionally, interplay of these drivers within and across the categories is demonstrated in the model [Fig. 1].

## Discussion

Dairy farming sector in India has seen a major turnaround in the last few decades. As a result of the “white revolution”, India now has the distinction of being the largest milk producer in the world with 53.77 million tonnes in year 2017–18 [16]. Interestingly, this growth has been supported by a production system that comprises of 70% milk producers being smallholders and landless households [17]. However, a large part (42%) of this increase in milk production has been attributed to increase in population of dairy animals, mostly crossbred cows [18]. Both of the above shifting trends increase vulnerability to infections in animals and risk of zoonoses to humans. This is especially true of intensive livestock systems, typically observed in peri-urban areas that struggle for space, infrastructure and programmatic oversight.

Knowledge of good animal husbandry practices, farm hygiene and clean milk production are critical for safe and sustainable milk production. Extension services are supposed to play an important role in imparting this knowledge on a regular basis. Similarly, organized systems of milk procurement and supply such as cooperatives can be an important source of knowledge and oversight of good practices. India, however, fails on both these accounts. Evidence suggests that public sector extension system is a source of information for about 10% farmers, with 33rd schedule of NSSO’s ‘Situation Assessment Survey of Framers’ revealing that 60% of farmer households in India had not accessed any information on modern technology in past 1 year [19]. Similarly, in spite of their tremendous growth, only 10% of farmers are associated with cooperative system in India [18]. Clearly, there appears to be an increased vulnerability to emergence, persistence and spread of infectious diseases in milk production systems in India.

This qualitative enquiry studied dairy farming practices in peri-urban small holder farms in three cities in India, each characterized by a different trading system, in the context of vulnerability to Btb. Btb is a zoonoses that has been widely underreported and understudied but is believed to be a significant contributor to animal and human losses in India [20, 21]. Majority dairy farmers in these settings were unaware about ‘tuberculosis’ in cattle. Farmers’ knowledge was limited to human tuberculosis and their symptoms and had strong perception that tuberculosis is a disease affecting ‘only’ humans. In general, in the absence of a clear mechanism in place for disposal of dead animals, the farmers engaged in selling of sick animals to minimize losses, once the resources and efforts to treat the animals had been exhausted. This practice was also applicable to cattle affected by Btb and could likely contribute to spread of infection to other animals as well as animal handlers. Lack of knowledge about the spread of disease in the herd and its zoonotic potential was also reflected in reliance on traditional knowledge without having a systematic screening system in place. Low level of knowledge related to zoonoses like bovine tuberculosis and bovine brucellosis and their zoonotic potential has been reported widely in a recent times [22–26]. Awareness about the disease and zoonotic potential is highly limited to commercial farmers with good access to resources. In a study in Zimbabwe, significantly higher percentage of commercial dairy farmers (65.0%) being aware compared to smallholder dairy farmers (36.7%) [27]. This could be attributed to lack of access to resources and service by extension department, a pattern that was also observed in our study.

At the system level, efforts directed at prevention and control of Btb were found to be grossly inadequate. Btb was not perceived as a problem and as result there was no system for regular screening of animals, except for voluntary testing by farmers or in more organized systems such as military and university farms. Expectedly, skin testing for Btb was found to be more of an academic exercise in university departments rather than a regular program feature. Efforts to increase awareness among farmers through outreach activities were also found to be absent. For example, few farmers in Bangalore reported that although they had attended seminars and workshop, tuberculosis was never discussed on these platforms. The Assistance to States for Control of Animal Diseases (ASCAD) programme provides a framework wherein states are free to choose the disease which, according to them, are important to their respective state [28]. Current narrative indicates that the choice of investment under ASCAD by the states is majorly dependent on the recent outbreaks in the financial year and on available burden estimates [28]. Btb is likely to be left out of this mechanism and in the absence of a push from the human health sector for its zoonotic significance the neglect is likely to persist. Hence there is an urgent need for the research community on both sides to invest in this area, generate evidence and support prevention and control efforts.

Limited appreciation of Btb as a problem could be the consequence of overall policy neglect, largely driven by lack of evidence. Qualitative narrative and the literature review show that bovine tuberculosis among cattle is one of the least studied disease with limited epidemiological evidences to support the decision making in India. Studies from South-East Asia reported animal level prevalence of bovine tuberculosis up to 24.7% [29, 30]. A recently published systematic review and meta-analyses on prevalence bovine tuberculosis considered 11 studies from past 10 years from India [9]. Out of these, four studies are from single Indian state. At the same time, anecdotal evidence of bovine tuberculosis among cattle, by the field veterinarians based on their clinical understanding continuously substantiates the need of epidemiologically strong empirical inquiries. Furthermore, the chronic nature of the disease that doesn’t present as acute outbreaks, doesn’t allow it to be perceived as a threat by farmers, programs and policy makers likewise. Considering the dearth of burden estimates, systematic scientific evidence making is required at national level to calculate the disease burden and identification of hot spots to address the issue and decision making. India could also make use of the surveillance systems established under NADRES (National Animal Disease Referral Expert System) to systematically generate evidence on burden of Btb – screening efforts using skin testing as well as direct evidence in post mortems [31]. This could be supplemented with periodic surveys.

Limited specific technical options available to policy makers and program managers for control of Btb, such as test and slaughter or test and isolation, could also explain the policy neglect. In India, cattle are considered sacred and slaughtering is constitutionally banned [32]. In light of the socio-cultural beliefs of the people, alternate strategies have to be figured out to control bovine tuberculosis. Infected cattle could be isolated from the herd and moved to a separate space. Farm level economics is an important factor in decision making. Isolation could be supplemented with the appropriate compensation to meet milk productivity loss.

Intensification of dairy production in the peri-urban fringes could potentially be helpful in alleviating the poverty among the dairy farmers which could further address the issue of risk practices like selling sick cattle for slaughtering through unregulated channels as well as in cattle fairs. Better economic condition would further enhance capacity to pay for veterinary consultations, medications etc. Improved feeding, especially better use of concentrate feed, well-developed marketing systems with processing infrastructure, improving genetic quality of the herd through support to private or co-operative-based AI services are some of the potential options for successful intensification of dairy sector in India [33, 34].

While a synergy emerged among the stakeholders as reflected in general lack of knowledge and appreciation of Btb as a problem, interesting divergent perspectives that are equally important in informing next steps, were worth noting. The animal husbandry department indicated towards an element of secrecy, over-reliance on traditional knowledge and avoidance of formal health care services on part of the farmers in the event of sickness in the herd. On the other hand, the health care system was perceived as severely constrained by the farmers in terms of providing knowledge, technical support, economic support and access in general. As a result, they had to rely on more predictable sources of information such as traditional knowledge, peers and private practitioners. Any efforts at system strengthening should therefore address the trust and credibility deficit for program impact.

### Limitations of the study

A very small number of state-level civic officials were involved in this study. Notwithstanding this, the limited number provided rich and meaningful data as the respondents who participated had decades of experience in animal husbandry and veterinary medicine. IDI with dairy farmers were performed in the local languages and then translated into English. Despite the rigorous verification process, some subtle nuances might have been missed during the verbatim transcribing.

## Conclusions

There are serious knowledge deficits and lack of appreciation of Btb as an animal and public health problem. While investments have lacked at the program and policy level, a large part could be attributed to lack of evidence and focus by the research community. The evidence is needed not only about the burden and risks, but also on possible options for control applied in the local Indian setting. Meanwhile, there is preliminary evidence that can at least inform initiation of awareness about the problem of Btb in animals, its health and economic impact in animals and zoonotic potential in humans. The response has to be initiated from both animal and human health sectors.

## Additional file

Additional file 1: Thematic Guides. Thematic guides used for formal In-Depth Interviews of the stakeholders under the study. (PDF 251 kb)

## Abbreviations

ASCAD: The assistance to states to control animal diseases
Btb: Bovine Tuberculosis
FMD: Foot and Mouth Disease
IDI: In-depth interviews
NADRES: National Animal Disease Referral Expert System
OIE: World Organization for Animal Health
RNTCP: Revised National Tuberculosis Control Programme
