# Supplementary material for: Community, system and policy level drivers of bovine tuberculosis in smallholder periurban dairy farms in India: a qualitative enquiry
Source: BMC Public Health. 2019 Mar 13;19:301. doi: 10.1186/s12889-019-6634-3 (PMC6415345; doi:10.1186/s12889-019-6634-3)
Supplement: Supplementary file 1 — Thematic Guides. Thematic guides used for formal In-Depth Interviews of the stakeholders under the study. (PDF 251 kb) [file 12889_2019_6634_MOESM1_ESM.pdf]

***Civic officials[corporation officials, PCB officials, city councillors]***

Personal Introduction-introduction of the research study- consent process

KI Introduction

- Could you tell us/me about your experience in the department/in professional field?
  [**Probes***: exact title, role and years of involvement*]

Sign Post [Use any clues given in introduction and sign post to next topic]

**Situation of peri-urban dairy farms**

- Can you tell me something about the dairy farms that exists in your city?
  [***Probes***: Type of farms and cattle species, locations, community involved in dairy farming]

- At present what is the value of small holding dairy farms in this city and its milk supply?
  [***Probes***: Value in providing milk/milk products for population]

- Can you tell me something about how does milk get supplied from dairy farms to end users in your city?
  [***Probes***: supply chain details, stakeholders/agencies involved in the supply chain]

- Can you tell me about the major health related issues that small holding dairy farmers and their cattle face?
  [***Probes***: Health risks to farmers, health risks to cattle, local risk mitigation strategies followed]

**Risks**: probe about the 6 levels of risks i.e., ***Ecosystem to cattle, Milke shed to cattle, Dairy to milk, milk handlers to milk, transport to milk, milk to consumer***

Sign Post [Summarise then use any clues given in this section and sign post to next topic]

**Infrastructure and support systems**

- What is the support that is currently provided to small holding dairy farmers in your city?
  [***Probes***: ***Types of support*** such as water supply, treatment and disposal of waste, medical services, ***departments involved*** in the provision of each service, ***cost of services*** provided, ***acceptability of services*** by the community]

- What are the challenges that you face in providing services to small holding dairy farmers in your city?
  [***Probes***: Challenges in supplying water, treatment and disposal of waste sanitation, medical support services]

- Can you tell me something about the regulation and milk and milk products in your city
  [***Probes***: Agencies involved, how is it implemented]

**Policy framework**

- Can you tell me something about any policy initatives to regulate and improve the condition of dairy farmers in urban centres?
  [***Probes***: ]

- What is the policy regarding antibiotic usage by dairy farmers in the state?
  [***Probes***: Policy origin, level of implementation, chalenges in implementation]

- Do you have any specific policy framework in place for the management of zoonotic diseases? (particularly Bovine TB)
  [***Probes***: stakeholders involved in the process of formulation, and implementation of policies, challenges in implementing the policy]

- From your experience do you have any suggestions to improve the conditions under which small holding dairy farms and farmers operate in your city?
  [***Probes***: Specific suggestions to improve health related issues of both farmers and cattle and risk reduction along the supply chain ]

**Conclusion**

Thank you very much for your time and for sharing about your experience with us. These are all our questions for now . Is there something important that we have missed out that you feel you would like to tell us from your experience?

In case we have any clarifications regarding what you have shared with us would it be alright to get in touch with you later on telephonically or in person depending on your convenience?

[if they elect to see transcript]: We hope to have the interview transcribed over the next ten days and I will send it to you by email at that time or if you prefer I can send you a copy of the transcript in English/local language by post. Can I have your contact details for this purpose?

***Community members [farm owners, managers, workers]***

Personal Introduction-introduction of the research study- consent process

Informant Introduction

- Could you tell us/me about how long you have been involved with dairy farming?
  [**Probe***s: , how did they get involved, exact position and years of involvement*]

Sign Post [Use any clues given in introduction and sign post to next topic]

**<u>Situation of peri-urban dairy farms</u>**

- Can you tell me something about your dairy farm?
  [***Probes***: Type of farm and species of cattle, no of cattle- details, vaccination status]

- Can you briefly desribe the major activities that happen in your farm?
  [**Probes**: Daily activities, people involved at the farm]

- Can you tell me something about how does milk produced on your farm get supplied to people?
  [***Probes***: Packaging details, customers/stakeholders/agencies involved in the supply chain, process of selling, payment details, challenges faced in selling milk if any]

- In addition to milk are there other products that are produced in your farm?
  [**Probes**: Supply chain details, stakeholders/agencies involved in the supply chain]

- Can you tell me about the major health related issues that you or your cattle have faced so far that?
  [***Probes***: Health risks to cattle, Health risks to self and workers]

<u>**Risks**: probe about the 6 levels of risks i.e., ***Ecosystem to cattle, Milke shed to cattle, Dairy to milk, milk handlers to milk, transport to milk, milk to consumer***</u>

- Can you tell me about some of the risk reduction strategies that you follow in your both for humans and cattle?
  [**Probes**: Guide participant with an example for each of the first four levels of risk above]

- How do you diagnose disease in your cattle?
  [**Probes**: Ways of diagnosis, use of lab tests, tests conducted]

- In case any animal is sick what do you do?
  [**Probes**: Treament practices that are being followed currently]

- What are the medicines that you use to treat sick cattle/buffaloes?
  [**Probes**: Names of medicines, preferred ones and why, are medicines added to the feed]

- Can you tell me something about the type of Lab support that you use/ get for diagnosing sick animals?
  [**Probes**: Source of lab support, most common tests carried out, costs involved, accessiblity of labs]

- How do you manage animals that are are very sick or die?
  [**Probes**: Practices that are being followed currently including disposal mechanisms and costs involved]

Sign Post [Summarise  then use any clues given in this section and sign post to next topic]

**Infrastructure and support systems**

- How to you access water and other services such as disposal of farm waste?
  [**Probes**: Details of current practise, cost and stakeholders involved in each, challenges faced in accessing services]

- What role does the extention departments of veterinary colleges/animal husbandry department play in ensuring the health of the animals on your farm?
  [**Probes**: Relationship between farmers and veterinary departments, accessibility of services, cost of services, value of services]

- What is the support that is currently provided to dairy farmers like you in your city?
  [***Probes***: ***Types of support*** such as water supply, treatment and disposal of waste, medical services, ***departments involved*** in the provision of each service, ***cost of services*** provided, ***acccesibility and usefulness of services*** provided]

Sign Post [Summarise  then use any clues given in this section and sign post to next topic]

**Community**

- Who are the dairy owners in this place?
  [**Probes**: Individuals or collective, community details]

- Are dairy owners organised in this place?
  [**Probes**: Cooperatives, unions, stand alone individuals]

- What are the major challenges to being a dairy farmer in your area?
  [**Probes**: economic viability, political issues, lack of support systems, future prospects for dairy farming]

**Conclusion**

Thank you very much for your time and for sharing about your experience with us. These are all our questions for now . Is there something important that we have missed out that you feel you would like to tell us from your experience?

In case we have any clarifications regarding what you have shared with us would it be alright to get in touch with you later on telephonically or in person depending on your convenience? [if they elect to see transcript]: We hope to have the interview transcribed over the next ten days and I will send it to you by email at that time or if you prefer I can send you a copy of the transcript in English/local language by post. Can I have your contact details  for this purpose?

***[Veterinarians/Veterinary departments]***

Personal Introduction-introduction of the research study- consent process

KI Introduction

- Could you tell us/me about your experience in the department/in professional field?
  [**Probes**: *exact title, role and years of involvement*]

Sign Post [Use any clues given in introduction and sign post to next topic]

**Landscape of peri-urban dairy farms**

- Can you tell me something about the dairy farms that exists in your city?
  [***Probes***: Type of farms and cattle species, locations, avg holding size, community involved in dairy farming]

- How are the dairy farmers in this city organised?
  [**Probes**: Cooperatives/unions- dominant ones, roles they play, political affiliation/significance of groups]

- Can you tell me about the major health related issues that small holding dairy farmers and their cattle face?
  [***Probes***: Health risks to farmers, health risks to cattle, local risk mitigation strategies followed]

- Who are the main providers of diagnostic and treatment services to dairy farms?
  [**Probes**: How are sick animals managed, first point of service provision, list of service providers in the area]

- How do farmers in your area manage animals that do not respond to treatment or die?
  [**Probes**: Local strategies adopted, support systems available]

**Risks**: probe about the 6 levels of risks i.e., ***Ecosystem to cattle, Milke shed to cattle, Dairy to milk, milk handlers to milk, transport to milk, milk to consumer***

Sign Post [Summarise then use any clues given in this section and sign post to next topic]

- Can you tell me something about how does milk get supplied from dairy farms to end users in your city?
  [***Probes***: supply chain details, stakeholders/agencies involved in the supply chain]

**Extension services and support systems**

- When we talk of extension services for dairy farms in your city what are the key services that are provided?
  [**Probes**: list (types) of extension services, main providers, what services do dairy farmers seek from you]

- What is the support that is currently provided by your department/clinic to small holding dairy farmers in your city?
  [***Probes***: ***Types of support*** including medical services, ***cost of services*** provided, ***acceptability of services*** by the community]

- What are the laboratory services that you provide dairy farms in your area?
  [**Probes**: Types of services, common tests carried out, challenges faced, others providing lab services]

- Do you test milk from sick cows?
  [**Probes**: Process- who initiates, test conducted, culture and sensitivity test and its challenges]

- We would like to ask you now about a specific test –have you done intradermal tuberculin test (purified protein derivative) on cattle in your city the last one year?

  [**Probes**:Frequency of using the test, response to animals who test positive, challenges in using the test] ***<u>If test was not done then probe why</u>***

- Can you tell me something about the regulation and milk and milk products in your city
  [***Probes***: Agencies involved, implementation of regulation]

- Can you tell us something about the use of medicines by dairy farmers in your area?
  [**Probes**: Types of medicines used and their availability, use in treament & feed additives, major illness for which medicine is used, challenges in regulating use]

- What are the challenges that you face in providing services to small holding dairy farmers in your city?
  [***Probes***: Challenges in access, providing diagnostic services, treament services, behaviour change among dairy farmers]

**<u>Policy framework</u>**

- Can you tell me something about any policy initatives that you are aware of in your state to regulate and improve the condition of dairy farmers in peri-urban centres?
  [***Probes***: Names of policy initatives and their features ]

- What is the policy regarding antibiotic usage by dairy farmers in the state?
  [***Probes***: Policy origin, level of implementation, chalenges in implementation]

- Do you have any specific policy framework in place for the management of zoonotic diseases? (particularly Bovine TB)
  [***Probes***: stakeholders involved in the process of formulation, and implementation of policies, challenges in implementing the policy]

- From your expereince do you have any suggestions to improve the conditions under which small holding dairy farms and farmers operate in your city?
  [***Probes***: Specific suggestions to improve health related issues of both farmers and cattle and risk reduction along the supply chain ]

**<u>Conclusion</u>**

Thank you very much for your time and for sharing about your experience with us. These are all our questions for now . Is there something important that we have missed out that you feel you would like to tell us from your experience?

In case we have any clarifications regarding what you have shared with us would it be alright to get in touch with you later on telephonically or in person depending on your convenience?

[if they elect to see transcript]: We hope to have the interview transcribed over the next ten days and I will send it to you by email at that time or if you prefer I can send you a copy of the transcript in English/local language by post. Can I have your contact details  for this purpose?
